# Supplementary material for: COZOID: contact zone identifier for visual analysis of protein-protein interactions
Source: BMC Bioinformatics. 2018 Apr 6;19:125. doi: 10.1186/s12859-018-2113-6 (PMC5889581; doi:10.1186/s12859-018-2113-6)
Supplement: Supplementary file 2 — Software build. Executable binary file of the software tool. (ZIP 118,784 kb) [file 12859_2018_2113_MOESM2_ESM.zip › cozoid/platform/docs/testng-6.5.1-javadoc.zip!/org/testng/ITestListener.html]

ITestListener (TestNG)


---


|  |  |  |
| --- | --- | --- |
| **Overview** \| **Package** \| **Class** \| **Tree** \| **Deprecated** \| **Index** \| **Help** | |  |
| **PREV CLASS**   **NEXT CLASS** | **FRAMES**    **NO FRAMES**     **All Classes** |  |
| SUMMARY: NESTED \| FIELD \| CONSTR \| METHOD | DETAIL: FIELD \| CONSTR \| METHOD |  |


---


## org.testng Interface ITestListener

**All Superinterfaces:**: ITestNGListener

**All Known Implementing Classes:**: TestListenerAdapter, TestNG.ExitCodeListener

---

``` public interface ITestListener extends ITestNGListener ```

A listener for test running.

**Author:**
:   Cedric Beust, Alexandru Popescu, Hani Suleiman

---

| **Method Summary** | |
| --- | --- |
| `void` | `onFinish(ITestContext context)`             Invoked after all the tests have run and all their Configuration methods have been called. |
| `void` | `onStart(ITestContext context)`             Invoked after the test class is instantiated and before any configuration method is called. |
| `void` | `onTestFailedButWithinSuccessPercentage(ITestResult result)`             Invoked each time a method fails but has been annotated with successPercentage and this failure still keeps it within the success percentage requested. |
| `void` | `onTestFailure(ITestResult result)`             Invoked each time a test fails. |
| `void` | `onTestSkipped(ITestResult result)`             Invoked each time a test is skipped. |
| `void` | `onTestStart(ITestResult result)`             Invoked each time before a test will be invoked. |
| `void` | `onTestSuccess(ITestResult result)`             Invoked each time a test succeeds. |

**Method Detail**

### onTestStart

```
void onTestStart(ITestResult result)
```

:   Invoked each time before a test will be invoked.
    The `ITestResult` is only partially filled with the references to
    class, method, start millis and status.

    :   **Parameters:**: `result` - the partially filled `ITestResult` **See Also:**: `ITestResult.STARTED`

---


### onTestSuccess

```
void onTestSuccess(ITestResult result)
```

:   Invoked each time a test succeeds.

    :   **Parameters:**: `result` - `ITestResult` containing information about the run test **See Also:**: `ITestResult.SUCCESS`

---


### onTestFailure

```
void onTestFailure(ITestResult result)
```

:   Invoked each time a test fails.

    :   **Parameters:**: `result` - `ITestResult` containing information about the run test **See Also:**: `ITestResult.FAILURE`

---


### onTestSkipped

```
void onTestSkipped(ITestResult result)
```

:   Invoked each time a test is skipped.

    :   **Parameters:**: `result` - `ITestResult` containing information about the run test **See Also:**: `ITestResult.SKIP`

---


### onTestFailedButWithinSuccessPercentage

```
void onTestFailedButWithinSuccessPercentage(ITestResult result)
```

:   Invoked each time a method fails but has been annotated with
    successPercentage and this failure still keeps it within the
    success percentage requested.

    :   **Parameters:**: `result` - `ITestResult` containing information about the run test **See Also:**: `ITestResult.SUCCESS_PERCENTAGE_FAILURE`

---


### onStart

```
void onStart(ITestContext context)
```

:   Invoked after the test class is instantiated and before
    any configuration method is called.

---


### onFinish

```
void onFinish(ITestContext context)
```

:   Invoked after all the tests have run and all their
    Configuration methods have been called.


---


|  |  |  |
| --- | --- | --- |
| **Overview** \| **Package** \| **Class** \| **Tree** \| **Deprecated** \| **Index** \| **Help** | |  |
| **PREV CLASS**   **NEXT CLASS** | **FRAMES**    **NO FRAMES**     **All Classes** |  |
| SUMMARY: NESTED \| FIELD \| CONSTR \| METHOD | DETAIL: FIELD \| CONSTR \| METHOD |  |


---
